# Effectiveness of Porcelain Polishing Methods on the Roughness and Color Stability of CAD–CAM Ceramics after Orthodontic Bracket Debonding

**DOI:** 10.3390/ma16144951

**Published:** 2023-07-11

**Authors:** Satheesh B. Haralur, Abdullah Saeed Shiban, Nasser Mohammed Alqahtani

**Affiliations:** 1 Department of Prosthodontics, College of Dentistry, King Khalid University, Abha 62529, Saudi Arabia; nmalqahtani@kku.edu.sa; 2 Interns, College of Dentistry, King Khalid University, Abha 62529, Saudi Arabia; abdullahshiban97@gmail.com

**Keywords:** surface roughness, porcelain color, lithium disilicate CAD, polymer-infiltrated ceramic, zirconia-reinforced lithium silicate glass ceramic, 5YTZP zirconia, orthodontic bracket debonding

## Abstract

Increased utilization of all ceramic restorations for aesthetic consideration in combination with routine adult orthodontic practice has led to numerous clinical challenges. Obtaining adequate bond strength between the orthodontic bracket and all-ceramic restoration and damage to the ceramic surface during the bracket debonding procedure needs to be better understood. This study analyzed the surface texture and color change of glazed and bracket-debonded ceramic CAD–CAM materials and the efficiency of porcelain polishing systems in restoring surface morphology and color. A total of 30 rectangular test specimens from each lithium disilicate CAD (LD-CAD), polymer-infiltrated ceramic (PICN), zirconia-reinforced lithium silicate glass ceramic (ZLS), and 5YTZP zirconia (5YZP) were prepared. The ceramic samples were embedded within acrylic resin, and baseline surface roughness and color were recorded using an optical profilometer and a reflected color spectrophotometer. The porcelain samples were bonded with lower incisor ceramic brackets. The samples were thermocycled 2000 times between 5 and 55 °C. Afterward, the brackets were peeled with bracket-removing pliers. The resin adhesive from the debonded surface was eliminated with tungsten carbide bur, and samples were randomly divided into three subgroups of (n = 10) to be polished with Diapol-twist (DT), a Keramik-Politur 4313B Komet polishing kit (KK), and a Horico diamond polisher (HDP). The polished surface roughness and color were recorded. The data were statistically analyzed using a two-way analysis of variance at *p* < 0.05. The debonded porcelain surfaces across the groups displayed significantly higher mean surface roughness (Ra) compared to glazed surfaces. The debonded 5YZP showed the least Ra at 0.661 (0.09), while the highest Ra was recorded by PICN at 4.057 (0.82). All of the polishing systems evaluated in the study significantly reduced the surface roughness. However, they were unsuccessful in regaining the surface topography of the glazed surface. The flexible discs (DT) produced the smoothest surface, followed by the diamond-impregnated rubber points (KK). Unpolished porcelain surfaces had a discernable mean color difference from glazed surfaces. Polished surfaces from the different polishing systems among the all-ceramic groups achieved a clinically acceptable mean color difference. Polishing debonded rough porcelain surfaces is imperative, and flexible discs performed better in all of the tested CAD–CAM ceramic materials.

## 1. Introduction

The need for orthodontic care has increased dramatically among individuals with one or more restored teeth in recent years [1]. Porcelain is a commonly used dental material for replacing missing teeth or restoring severely damaged teeth and deteriorating enamel surfaces. This is due to its excellent strength and durability, excellent aesthetics, and acceptable biocompatibility [2]. Computer-aided design/manufacturing technology (CAD–CAM), which enables the fabrication of monolithic restorations with significant advantages in terms of aesthetics, economy, and processing time, has greatly expanded the possibilities of prosthetic restorations. Material innovation aiming to achieve optimal bio-mechanical properties analogous to tooth substrates led to the development of numerous materials such as lithium disilicate (LD-CAD), zirconia-reinforced lithium silicate glass ceramic material (ZLS), hybrid polymer-infiltrated ceramic (PIC), and 5YTZP zirconia (5YZP) ceramics. Lithium disilicate CAD is known for its superior color parameters, efficient bonding to the tooth structure, and high fracture strength of 2665.4 ± 759.2 N [3]. Zirconia oxide at 0.1 wt% was added to the glass–ceramic matrix to enhance biaxial strength up to 541 MPa in ZLS ceramics [4]. Meanwhile, hybrid PIC was developed to obtain the combined material benefit of ceramic (60–86%) and polymer (40–14%) and develop the compatible modulus of elasticity of dentin [5]. The 5YZP ceramics had the distinct advantage of high flexural strength (500–800 MPa) combined with smooth color progression and no discernible layer [6].

Following orthodontic treatment, the goal of debonding is to eliminate orthodontic attachments and residual adhesives from the tooth and restorations, as well as to bring the restoration or tooth surface back to its original state as closely as possible [7]. However, the debonding process is often reported to result in iatrogenic irreversible damage to the enamel and restoration surface, including surface fractures, vertical cracks, and roughness [8]. It is also essential to remove residual adhesive resin after bracket debonding. Adhesive resin removal is attempted using various methods, including manual scalers, orthodontic band removal pliers, rotary diamond or tungsten carbide burs, ultrasound, aluminum oxide air abrasion, and lasers. The resultant rough surface promotes plaque buildup, periodontal disease, porcelain discoloration, and reduced mechanical properties. Large surface irregularities and roughness also adversely affect porcelain color due to less light reflection than glazed surfaces [9]. Improper debonding and clean-up procedures may lead to enamel damage that could permanently affect tooth structure [10]. Debonding and adhesive removal are even more challenging on concave dental surfaces when lingual appliances are used [11]. This could result in a greater amount of enamel damage due to the difficulty in adhesive removal. Typically, glazing porcelain increases its smoothness and resistance to fractures and reduces wear by filling its pores. However, removing and re-glazing ceramic restorations is challenging, more complicated, and time-consuming. Ceramic microstructures may also be affected by the glaze process, such as devitrification.

As an alternative, polishing can be performed chairside and requires only one clinical session. Research has been conducted on porcelain polishing techniques to find the most efficient method for smooth porcelain surfaces. Some authors [12,13] recommend polishing rather than glazing, observing that hand polishing produces a smoother surface comparable to oven glazing. Ferracane [14] reported the superior surface texture of manually polished CAD–CAM ceramics compared to glazed surfaces. Nevertheless, a few researchers have concluded that polishing systems cannot reproduce smooth surfaces like glazed surfaces [15]. Intraoral techniques such as diamond burs and rubber polishers are generally utilized to obtain clinically smooth surfaces. Few researchers have found diamond polishing paste to be more effective than polishing stones [16]. Studies have demonstrated that Sof-Lex discs effectively reduce porcelain surface roughness after glazing [17].

A few manufacturers have introduced specific polishing systems, yet not all dental ceramic manufacturers provide polishing systems, and clinicians must determine the suitable systems for various CAD–CAM ceramics. The polishability of ceramics and the effectiveness of polishing systems are affected by fillers, crystal size, glass matrix, and compositions. There is a paucity of dental literature regarding the effect of different polishing systems on the surface morphology and color parameters of various debonded CAD–CAM porcelain materials. Thus, the purpose of this study was to determine the efficacy of different polishing systems on the surface roughness and color change of debonded CAD–CAM porcelain surfaces. The first null hypothesis was that ceramic polishing systems produce roughness equivalent to baseline and there are no variations in surface texture among ceramic polishing systems. The second null hypothesis was that there is no difference in color parameters between glazed and polished ceramic surfaces.

## 2. Materials and Methods

**Figure 1:**
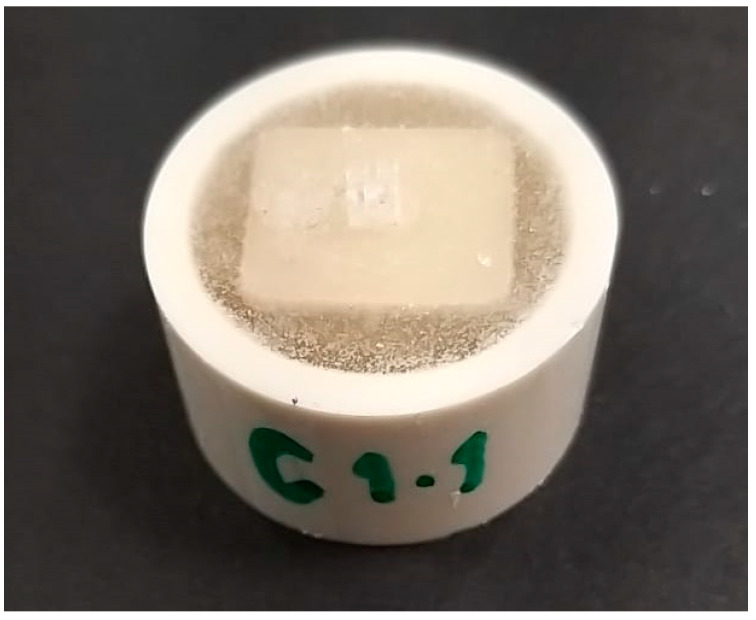
Ceramic disc sample with an attached orthodontic bracket.

Four CAD–CAM ceramic materials were tested in this in vitro study: Lithium disilicate (IPS e.max CAD; Ivoclar Vivadent AG, Germany), polymer-infiltrated ceramic (Vita Enamic, Vita Zahfabric, Bad Säckingen, Germany), zirconia-reinforced lithium silicate glass ceramic (Vita Suprinity, Vita Zahfabric, Bad Säck-ingen, Germany), and 5YTZP zirconia (Cercon XT; Dentsply Sirona, Charlotte, NC, USA) (Table 1). The samples from each CAD–CAM material were sectioned at 6 × 3 × 2 mm with a low-speed diamond saw (Isomet 1000, Buehler) under running water. e.max CAD glazing paste was evenly applied to LD-CAD ceramic samples using a small brush. The samples were then subjected to combined crystallization/glaze firing at 820–840 °C. The ZLS ceramic samples were manually polished with a diamond-coated pink pre-polishing cup at 10,000 rpm, followed by a grey diamond-coated high glass cup at 8000 rpm. The sintered 5YTZP zirconia crowns were polished sequentially with 320, 600, and 1200 grit SiC sandpaper under slow-running water on a rotary polisher (Jean Wertz, Charlottenstr. 73, Düsseldorf, Germany). Zirconia samples were then glazed with high-flu universal overglaze. The PIC ceramic discs were processed initially by pre-polishing with pink polishers at 10,000 rpm under light pressure and high-gloss polish with grey polishers at 8000 rpm. Ceramic disc was placed in a chlorinated PVC cylinder containing transparent self-curing poly(methyl methacrylate) acrylic resin (Major.Base.20, Major Prodotti Dentari S.p.A., Moncalieri, Italy) (Figure 1).

### 2.1. Ceramic Disc Groups

Forty samples of each ceramic type were fabricated. They were randomly divided into four subgroups (n = 10) according to the polishing systems utilized to finish the ceramic surface after debonding the porcelain brackets. The polishing systems used in the study were Diapol-twist (DT; EVE Ernst Vetter GmbH, Neureutstr, Keltern, Germany) Keramik-Politur (KK; Komet Dental, 3042 Southcross Blvd Rock Hill, SC, USA), and Horico (HDP; Hopf, Ringleb & Co. GmbH & CIE, Gardeschützenweg, Berlin, Germany).

According to previous studies, the sample size for each sub-group was set at 10 [18]. The sample size was selected using G* Power software (version 3.1, University of Düsseldorf) with effect sizes (d) of 1.4, 0.05, and 1-β (power) of 0.80 [19]. The average surface roughness values of the glazed and polished surfaces were used to estimate the effect size [17].

### 2.2. Ceramic Bracket Bonding and Debonding

Ceramic brackets (Roth E-Sapphire, Tac Eksen, Huzhou, Zhejiang, China) for the lower incisors (10 torque, 00 angulation, and a width of 2.55 mm) were bonded onto the CAD–CAM ceramic specimen according to standard protocols and prepared by a single operator. Two coats of silane coupling agent (RelyX Ceramic Primer, 3M ESPE, St. Paul, MN, USA) were applied and allowed to dry for 60 s. The bracket base was covered in a light cure composite (Transbond XT, 3M Unitek, St. Paul, MN, USA) and pressed onto the ceramic disc to remove extra material. Subsequently, the excess luting composite was removed with a periodontal probe. The composite resin was polymerized by 1200 mW/cm^2^ LED light (Bleuphase, Ivoclar Vivadent, Schaan, Lichtenstein) for 20 s. Rectangle-shaped ceramic samples were stored in distilled water at ambient temperature for 24 h and thermocycled 2000 times between 5 and 55 °C. Subsequently, the brackets were debonded with bracket-removing pliers (Hu-Friedy Mfg. Co., LLC Rockwell St. Chicago, IL, USA) by the peeling method to minimize ceramic surface damage. The remaining composite resin over the ceramic surface was removed by a 30-bladed finishing tungsten carbide bur (Dentsply Sirona, Charlotte, NC, USA) by a single operator. It was performed under dental loupe 2.5× magnification (Carl Zeiss AG, Oberkochen, Germany) until a visually smooth surface was achieved.

### 2.3. Ceramic Surface Polishing

Group -1 ceramic samples were finalized using a Diapol-twist (DT) ceramic polishing kit (RA-306). The polishing method involved using diamond grit twists of various coarseness levels, ranging from rough to fine. These twists were applied in a sequential manner, following a three-step procedure for the polishing process. Ceramic surface polishing was performed as per the manufacturer’s recommendations with 10,000 min^−1^/rpm under water coolant. The surface was smoothed by successively using twists of various coarseness levels for 60 s. Group-2 samples were modified and polished with a Keramik-Politur Komet (KK) polishing kit (4313B). The ceramic polishing kit included rubber polishers interspersed with diamond grits. The ceramic discs were polished according to the manufacturer’s instructions in a low-speed handpiece at 6000 min^−1^/rpm under water coolant. Ceramic polishers were sequentially used from coarse, medium, and finishing grit, each for 60 s.

Group-3 ceramic discs were polished using a Horico diamond polisher (HDP; W9664). The samples were polished for 60 s at 6000 min^−1^/rpm under water coolant.

### 2.4. Surface Roughness and Color Measurement

The specimens were cleaned for 10 min in an ultrasonic cleaning apparatus using deionized water, and then air-dried for 20 s. Surface roughness and CIELAB color parameters were measured at three stages: After glazing (before bracket bonding), on the debonded surface (after cleaning with a TC bur), and after polishing.

A Contour GT-K 3D Optical Profilometer (Bruker, Rudolf-Plank-Str. 27 Ettlingen Germany) was used to characterize and image the surfaces using 3D non-contact surface metrology. Vertical scan interferometry was used to measure samples using a 5× Michelson magnification lens with a field of view of 1 × 1 mm, a Gaussian regression filter, a scanning speed of 1×, and thresholding of 4. Samples were put on the platform and manually moved to produce an image on the microscope’s monitor. An interferometer that uses a white broadband light source was employed to measure the object’s surface roughness, as well as the difference in pixel height between adjacent pixels wider than 135 nm. The roughness (Ra) value of each sample was calculated by scanning it three times and averaging the results.

CIELAB color was determined by a spectrophotomer (Lab Scan XE, Hunter Associates Laboratory, Inc. Sunset Hills Road, Reston, VA, USA). The lab Scan XE spectrophotometer works on a light wavelength ranging from 400 to 700 nm with a large measurement diameter of 50 mm and measures the reflected color using 0°/45° geometry. The alteration in color resulting from bracket attachment, debonding, and re-polishing was computed using the formula: ΔE = ([ΔL*]2+ [Δa*]2+ [Δb*]2) × ½ [20]. ΔE = 3.5 units was considered a visually perceptible threshold level [21,22].

Scanning electron microscope images (JOEL, JSM-6610, Dearborn Rd, Peabody, MA, USA) of debonded ceramic surfaces and polished with different methods were obtained at 100× magnification for qualitative analysis.

### 2.5. Statistical Analysis

The data were analyzed with SPSS (version 23, IBM, Armonk, NY, USA). Adherence to the standard distribution was assessed by the Shapiro–Wilk test. The difference between Ra values and color was statistically evaluated with two-way analysis of variance. The significance level was kept at *p* < 0.050.

## 3. Results

Table 2 displays the mean Ra and Rq values, along with the standard deviation of the CAD–CAM porcelain materials before and after debonding and surface re-polishing with various polishing systems. Orthodontic bracket debonding significantly increased the mean surface roughness values of all porcelain groups. The significant increase in Ra values after debonding was observed in PIC from 0.575 ± 0.07 to 4.057 ± 0.82 µm, whereas the least enhancement in surface roughness was recorded in 5YZP from 0.234 ± 0.03 to 0.661 ± 0.09 µm. All of the porcelain polishing techniques used in this study facilitated the substantial reduction in Ra values, although not as effectively as the glazed surface. The highest Ra value (2.38 ± 0.11 µm) was recorded in the PIC group polished from HDP, and the lower Ra value (0.196 ± 0.01) was recorded in the LD-CAD group polished with DT.

The Rq (root mean square roughness) values followed a similar trend of Ra values with a substantial increase after bracket debonding. The DT, KK, and HDP polishing systems reduced the Rq values compared to debonded surfaces. The highest Rq values of 5.80 ± 0.77 µm were observed in the PIC groups, while the lowest mean Rq was recorded by 5YZP ceramic at 1.014 ± 0.14 µm. Post-polishing DT produced the lowest Rq on LDS-CAD at 0.540 ± 0.04 µm, followed by 5YZP with the KK polishing kit at 0.729 ± 0.04 µm. Table 3 shows the mean color changes from the glazed surface. The debonded surfaces in all of the tested ceramic groups displayed greater ΔE values than the polished surfaces. The highest ΔE value amongst the debonded surfaces was recorded by PIC (7.85 ± 1.31), followed by LD-CAD (4.36 ± 1.43), ZLS (3.84 ± 1.70), and 5YZP (3.52 ± 1.28) ceramics. Among the polished groups, the lowest ΔE was recorded by 5YZP polished with DT, while the highest ΔE was obtained by LD-CAD treated with HDP. A two-way ANOVA (Table 4) was performed to assess the influence of porcelain type and polishing system on the mean surface roughness. The results indicated a significant (0.000) main effect for porcelain type, F(3,180) = 750.28, *p* = 0.000; a significant main effect for polishing system, F(4,180) = 270.44, *p* = 0.000; a significant interaction between porcelain type and polishing system, F(12,180) = 50.38, *p* = 0.000. The two-way analysis of variance (Table 4) results demonstrated that ΔE showed significant differences by both porcelain type (*p* = 0.001) and polishing system (*p* = 0.000). A significant interaction was also observed between the porcelain type and polishing system, F(9,144) = 13.53, *p* = 0.000.

**Figure 2:**
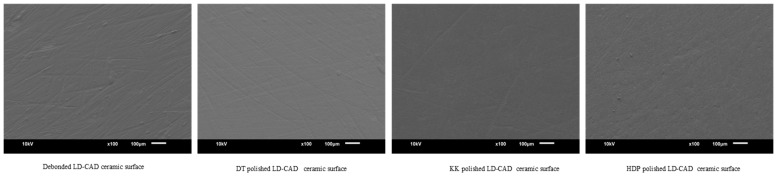
SEM images of LD-CAD ceramic debonded and polished surfaces.

**Figure 3:**
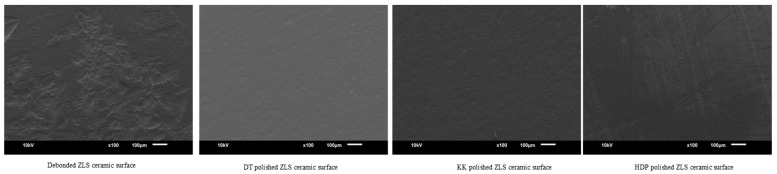
SEM images of ZLS ceramic debonded and polished surfaces.

**Figure 4:**
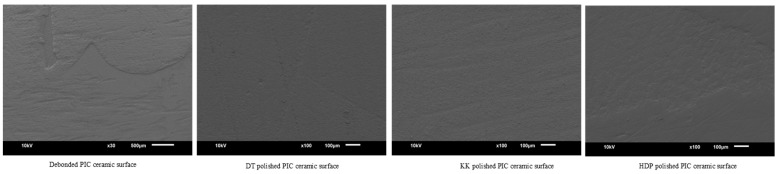
SEM images of PIC ceramic debonded and polished surfaces.

**Figure 5:**
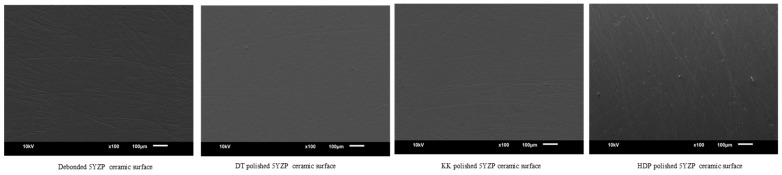
SEM images of 5YZP ceramic debonded and polished surfaces.

SEM images of debonded ceramic surfaces and polished using different methods are displayed in Figure 2, Figure 3, Figure 4 and Figure 5. The SEM images of unbonded ceramic surfaces displayed large irregular and deep grooves.

## 4. Discussion

Following the debonding of an orthodontic bracket, it is critical to polish the porcelain surface. An unpolished porcelain surface impairs aesthetics, encourages plaque buildup, and reduces porcelain durability. The restoration or natural teeth surface roughness exceeding 0.2 μm leads to enhanced plaque accumulation. Hence, Bolen et al. [23] recommended a mean surface roughness lower than 0.2 μm. Numerous studies have reported intact enamel surface roughness to be around 0.64 μm [24]. An unduly rough restoration surface affects the patient’s comfort, especially perceptive to the tongue. Jones et al. [25] suggested an Ra value threshold of 0.5 µm for patient amenity. In addition, rough porcelain surfaces lower the material’s flexural strength, making it more prone to fracturing at lower occlusal forces. Unlike glazed surfaces, it is susceptible to discoloration by various stains [26].

The current in vitro investigation compared the surface properties and color parameters of four different CAD–CAM ceramic materials before and after orthodontic bracket debonding and polishing. Due to the variations in surface roughness and color parameters with the polishing method, the null hypothesis that polishing systems do not alter surface roughness and color parameters was rejected.

The study results showed higher Ra and Rq values on the debonded porcelain surface compared to the glazed surface in all tested groups. LD-CAD porcelain increased the Ra from 0.85 (0.02) to 1.445 (0.20). Similar trends were observed in the ZLS, PIC, and 5YZP groups. The increase in surface roughness may be due to surface modification during the bonding process and composite left over on the debonded surface. Shear forces in excess of 13 MPa during debonding can lead to fracturing of the ceramic surface [27]. Orthodontic brackets and the subsequent removal procedure result in an irreversible change in surface morphology regardless of porcelain type [28]. Abrasive stones can be used to remove irregularities from porcelain surfaces, but they cannot restore the original surface. A significant difference in surface roughness between pre-bonded and debonded surfaces has been confirmed by several authors [15,29].

Reports on the outcome of various polishing systems are conflicting. Bourke et al. [30] noted that debonded feldspathic porcelain surface parameters can be restored by polishing. Sarac et al. [31] reported that felspathic porcelain is as smooth as glazed after polishing with a porcelain adjustment kit alone or preceding polishing paste. However, Steiner et al. [32] concluded that none of the commonly used ceramic polishing kits can create a surface smoother than that of glazed ceramic. Several factors may account for the contradictory results, including the bonding protocol, bracket type, bracket debonding force, and polishing system.

The present study results indicate that the diamond-impregnated spiral finishing discs used sequentially from smoothing, pre-polishing, and high-shine polishing twists produced the best results across all of the ceramic groups. The LD-CAD polished with Eve-twist resulted in a pre-bonded surface of Ra = 0.196 compared to Ra = 0.185. Similar results were reported by Sasany et al. [29], Flury et al. [33], and Karan et al. [34]. Their study, which involved feldspathic porcelain, leucite, and lithium disilicate ceramic polished with two different polishing kits, concluded that polishing techniques affect the surface roughness more significantly than the porcelain type. They reported that, in comparison to polishing wheels, Sof-Lex discs produce a finer and smoother surface. Pores incorporated within the porcelain body during the fabrication process tend to get exposed once the surface glaze is removed [35]. Osorio et al. [36] assessed the six polishing systems for residual resin removal and reported the Sof-Lex discs produce a smooth surface. Flexible polishing discs are advantageous in polishing flat and convex surfaces [37]. Our study report also recorded that cone- and flame-shaped polishers result in a coarser surface than a flexible polishing twist. Kou et al. [38] reported similar results of improved flexible disc performance compared to diamond-impregnated polishing systems.

Microstructure greatly affects ceramic polishing performance. A few ceramics are easier to polish than others, which is likely related to the composition of the glass matrix. In IPS e.max CAD, crystalline lithium disilicate filler is 70 vol% in a ceramic glass matrix. Regular surface topography is achieved by polishing this dense composition [24].

The PIC ceramic displayed higher Ra values in both debonded and polished surfaces. Surfaces of resin-based ceramics may be rougher due to higher filler content, smaller particles, or individual material properties [36]. Additionally, PIC ceramics with polymer matrix content are predisposed to water absorption and degradation during the thermocycling process [39]. Siddanna [40] reconfirmed the effectiveness of spiral polishers on resin-based ceramics. Rubber cup polishers are not effective due to the limited rounding of protruded filler particles and the abrading of a soft matrix [41]. The ZLS ceramic groups displayed marginally higher Ra values compared to LD-CAD; similarly, flexible discs provided a smoother surface followed by rubber cups (KK). Shibasaki et al. [42] reported the same results with ZLS ceramics with a higher Ra than LD-CAD, while unpolished surfaces exhibited greater Ra than rubber cup polished groups. Matzinger et al. [43] illustrated rounded nano- and medium-sized filler particles in ZLS. Hence, they achieved better polishing results and lower surface roughness. The surface roughness of polished 5YZP ceramics was close to pre-bonded surfaces, and the Ra values between different polishing systems were marginally different. Polishing the roughened surface from coarse grit diamond bur is imperative to contain the phase transformation and prevent zirconia flexural strength decrease [44]. Sampaio-Fernandes et al. [45] reported that universal polishing set spiral-shaped polishers produce smoother surfaces than material-specific pointed polishers. According to Jum’ah et al. [46], there was a significant increase in Ra in ground 5YZP, and polishing protocols had a significant reduction in Ra.

The findings of our study indicate that debonded surfaces had higher mean color changes (ΔE) than glazed surfaces. The ΔE values for ZLS (3.84 ± 1.70) and 5YZP (3.52 ± 1.28) ceramics were in a similar range, with LD-CAD ceramic samples having a marginally higher ΔE of 4.36 ± 1.43. However, a large ΔE of 7.85 ± 1.31 was recorded by debonded PIC ceramics. Prior studies [47,48] have indicated that color changes below 3.5 are imperceptible and deemed acceptable in a clinical setting. The reflection of random specular light localized to the surface increases as the surface roughens. As a result, the surface appears brighter. Surface roughness has a significant impact on the L* value, the specularly reflected light component. The color shifts toward yellow as the b* value rises [49].

Microstructure, grain size, and porosity are key parameters that can greatly affect ceramic mechanical and optical properties. The increased color change in PIC could be attributed to its porous microstructure infiltrated by the polymer. They are more susceptible to thermal degradation during thermocycling, and the TEGDEMA component of the polymer absorbs water. The polished surface displayed less color change, with the results being in agreement with Sarıkaya et al. [50], who observed better color stability with less surface roughness. In the absence of an understanding of the magnitude of the color difference that is visually detectable, merely determining color differences between two objects is of little clinical value. Many researchers have studied both the perceptibility threshold and the clinical acceptability threshold of dental restorations. There is clearly a lack of consensus on both perceptibility and acceptability thresholds in the dental literature. Seghi et al. [51] and Ruyter et al. [47] reported a DE* value of 1 as visually detectable. In terms of the acceptability threshold, it ranges from 2.0 to 4.0. However, the majority of research [52,53] has determined its value as 3.7. Orthodontic bracket debonded surfaces showed ΔE values well above 3.7, except for 5YZP ceramics at 3.52, indicating clinically unacceptable color alteration. All of the polishing methods employed in this study were successful in reducing ΔE values to a level below the acceptable threshold. The Diapol-twist produced the lowest E value in 5YZP, while Keramik-Politur Komet resulted in the lowest E value in PIC ceramics. We found no significant differences between flexible polishing discs (DT) and diamond-impregnated rubber cups (KK) on ceramic color alteration. Moradinezhad et al. [54] also reported that the mean ∆E value with different surface treatment methods was below the acceptable threshold. Sasany et al. [29] recommended polishing with the Sof-Lex system and opinioned that the smoothness and color stability are material and surface treatment dependent. Ameli et al. [55] reported that debonded surface polishing with flexible discs results in a porcelain color closer to the baseline.

The clinical implication of the study results is that orthodontic bracket debonded ceramic surfaces are extremely rough. Hence, it is imperative for the dentist to polish debonded ceramic surfaces. This is to prevent the associated risks of increased plaque accumulation, staining, and reduced mechanical properties. Polishing also enables the debonded surface to reduce color mismatch from glazed surfaces. This study provided preliminary results that could be expanded to assess the long-term clinical performance of polishing. Simulation of ceramic wear can be useful for assessing the effect of polishing using a brushing simulation machine. It is necessary to conduct future studies to evaluate effective polishing methods for different surface treatments conducted over the porcelain surface during the adhesive procedure. Besides improving the surface morphology and roughness, grinding and polishing can also increase strength and age resistance. Surface stress is formed when the material is ground or polished, and if this adjustment process is extended, the depth stress increases, resulting in a decrease in flexural strength. Phase transition from tetragonal to monoclinic phases determines the degree of flexural strength reduction in zirconia ceramics. Hence, the effect of polishing methods on mechanical and thermal properties needs further studies. This in vitro study was limited by the fact that the samples were not cemented to teeth during color change evaluation. It is imperative that the in vitro results be correlated with clinical application. Furthermore, this study was not able to replicate intraoral conditions using saliva during the in vitro experiment.

## 5. Conclusions

Within the limitations of this in vitro investigation, the following conclusions were drawn: Orthodontic bracket removal results in a rough surface; it is essential to polish the debonded surface, and ceramic polishing systems significantly improve porcelain surface smoothness. However, it is not equivalent to a glazed surface. A flexible disc polishing system creates better surface smoothness than rubber cup polishers. The ceramic polishing process reduces the discernable color difference between debonded and glazed surfaces, producing the ΔE within the clinically acceptable range.

## Figures and Tables

**Table 1:** Ceramic materials used in the study.

Material	Composition	Manufacturer	Lot No.
Lithium disilicate (e.max^®^CAD)	SiO_2_, Li_2_O, K_2_O, P_2_O_3_, ZrO_5_, ZnO, Al_2_O_3_, MgO	Vita Zahnfabrik, Bad Säckingen, Germany	X15367
Hybrid polymer-infiltrated ceramic (Enamic)	UDMA, TEGDMA, SiO_2_, Al_2_O_3_, Na_2_O, K_2_O, B_2_O_3_, Zr_2_O, CaO	Vita Zahnfabrik, Bad Säckingen, Germany	59882
Zirconia-reinforced glass ceramic (Suprinity)	SiO_2_, Li_2_O, K_2_O, P_2_O, Al_2_O_3_, ZrO_2_, CeO_2_, La_2_O	Vita Zahnfabrik, Bad Säckingen, Germany	58081
5YTZP zirconia (Cercon xt)	DeguDent GmbH, Hanau-wolfgang, Germany	Dentsply Sirona	18043031

**Table 2:** Mean ± SD Ra and Rq (µm) values for porcelain ceramic surfaces before and after debonding and polishing with statistical summaries.

Group	Test Results	Before Bonding	Debonded	Polished		
				DT	KK	HDP
LD-CAD	Ra	0.185 ± 0.02	1.445 ± 0.20	0.196 ± 0.01	0.368 ± 0.03	0.379 ± 0.02
	Rq	0.497 ± 0.05	2.136 ± 0.27	0.540 ± 0.04	0.773 ± 0.06	0.925 ± 0.04
ZLS	Ra	0.306 ± 0.02	1.278 ± 0.38	0.457 ± 0.05	0.590 ± 0.04	0.628 ± 0.05
	Rq	1.390 ± 0.46	2.560 ± 0.56	1.200 ± 0.12	1.451 ± 0.15	1.679 ± 0.22
PIC	Ra	0.575 ± 0.07	4.057 ± 0.82	1.772 ± 0.14	2.192 ± 0.15	2.380 ± 0.11
	Rq	2.627 ± 0.20	5.800 ± 0.77	2.680 ± 0.16	3.268 ± 0.30	3.248 ± 0.26
5YZP	Ra	0.234 ± 0.03	0.661 ± 0.09	0.300 ± 0.04	0.305 ± 0.03	0.385 ± 0.71
	Rq	0.493 ± 0.02	1.014 ± 0.14	0.745 ± 0.05	0.729 ± 0.04	0.903 ± 0.05

LD-CAD: Lithium disilicate; ZLS: Zirconia-reinforced lithium silicate glass ceramic; PIC: Hybrid polymer-infiltrated ceramics; 5YZP: 5YTZP zirconia; DT: Diapol-twist; KK: Keramik-Politur Komet; HDP: Horico diamond polisher.

**Table 3:** Mean ± SD color changes of the ceramic samples from the baseline (glazed) surface and after debonding and surface polishing.

Group	Debonded	DT	KK	HDP
LD-CAD	4.36 ± 1.43	2.12 ± 1.15	1.59 ± 1.09	2.54 ± 1.40
ZLS	3.84 ± 1.70	1.73 ± 0.26	2.21 ± 0.56	2.47 ± 0.75
PIC	7.85 ± 1.31	1.45 ± 0.41	0.91 ± 0.30	1.47 ± 0.36
5YZP	3.52 ± 1.28	0.87 ± 0.20	1.90 ± 0.65	1.82 ± 0.42

**Table 4:** Two-way ANOVA analysis of Ra and ΔE mean color change values with different CAD–CAM ceramic and polishing systems.

Test	Resource	SS	Df	MS	F	*p*
Ra	Porcelain	108.130	3	36.043	750.281	0.000 *
	Polishing	51.968	4	12.992	270.443	0.000 *
	Porcelain * Polishing	29.043	12	2.420	50.380	
ΔE	Porcelain	20.408	3	6.803	5.480	0.001 *
	Polishing	344.760	3	114.920	92.585	0.000 *
	Porcelain * Polishing	121.827	9	13.536	10.906	0.000 *

Ra: Arithmetic mean roughness; ΔE: Mean color change; SS: Sum of squares; MS: Mean square. * Significant at the 0.01 level.

## Data Availability

Not applicable.

## References

[1] Bach G.K.G., Torrealba Y., Lagravère M.O. (2013). Orthodontic bonding to porcelain: A systematic review. Angle Orthod..

[2] Datla S.R., Alla R.K., Alluri V.R., Babu J.P., Konakanchi A. (2015). Dental ceramics: Part II-Recent advances in dental ceramics. Am. J. Mater. Eng. Technol..

[3] Baldissara P., Llukacej A., Ciocca L., Valandro F.L., Scotti R. (2010). Translucency of zirconia copings made with different CAD/CAM systems. J. Prosthet. Dent..

[4] VITA-Zahnfabrik. VITA SUPRINITY^®^ Technical|Scientific Documentation, VITA SUPRINITY^®^ PC-Glass Ceramic. Revolutionized. vita-zahnfabrik.com.

[5] Swain M., Coldea A., Bilkhair A., Guess P. (2015). Interpenetrating network ceramic-resin composite dental restorative materials. Dent. Mater..

[6] Mao L., Kaizer M.R., Zhao M., Guo B., Song Y.F., Zhang Y. (2018). Graded ultra-translucent zirconia (5Y-PSZ) for strength and function-alities. J. Dental Res..

[7] Arhun N., Arman A. (2007). Effects of Orthodontic Mechanics on Tooth Enamel: A Review. Semin. Orthod..

[8] Tholt B., Miranda-Júnior W.G., Prioli R., Thompson J., Oda M., Beatriz Tholt B., Tholta W.G., Miranda-Júniorb R., Priolic J.T., Odae M. (2006). Surface Roughness in Ceramics with Different Finishing Techniques Using Atomic Force Microscope and Profilometer. Oper. Dent..

[9] Tuncdemir A.R., Dilber E., Kara H.B., Ozturk A.N. (2012). The Effects of Porcelain Polishing Techniques on the Color and Surface Texture of Different Porcelain Systems. Mater. Sci. Appl..

[10] Paolone G., Mandurino M., Baldani S., Paolone M.G., Goracci C., Scolavino S., Gherlone E., Cantatore G., Gastaldi G. (2023). Quantitative Volumetric Enamel Loss after Orthodontic Debracketing/Debonding and Clean-Up Procedures: A Systematic Review. Appl. Sci..

[11] Paolone M.G., Kaitsas R., Paolone G., Kaitsas V. (2008). Lingual orthodontics and forced eruption: A means for osseous and tissue regeneration. Prog. Orthodont..

[12] Junior O.O., Buso L., Fujiy F.H., Lombardo G.H.L., Campos F., Sarmento H.R., Souza R. (2013). Influence of polishing procedures on the surface roughness of dental ceramics made by different techniques. Gen. Dent..

[13] Kaizer M.R., de Oliveira-Ogliari A., Cenci M.S., Opdam N.J., Moraes R.R. (2014). Do nanofill or submicron composites show improved smoothness and gloss? A systematic review of in vitro studies. Dent. Mater..

[14] Ferracane J.L., Broome J.C., Hilton T.J. (2013). A Contemporary Approach. Fundamentals of Operative Dentistry.

[15] Chu F.C., Frankel N., Smales R.J. (2001). Surface roughness and flexural strength of self-glazed, polished, and reglazed In-Ceram/Vitadur Alpha porcelain laminates. Int. J. Prosthodont..

[16] Winchester L. (1991). Direct Orthodontic Bonding to Porcelain: An In Vitro Study. Br. J. Orthod..

[17] Rashid H. (2012). Evaluation of the surface roughness of a standard abraded dental porcelain following different polishing techniques. J. Dent. Sci..

[18] Yamockul S., Thamrongananskul N., Poolthong S. (2016). Comparison of the surface roughness of feldspathic porcelain polished with a novel alumina-zirconia paste or diamond paste. Dent. Mater. J..

[19] Faul F., Erdfelder E., Buchner A., Lang A.-G. (2009). Statistical power analyses using G*Power 3.1: Tests for correlation and regression analyses. Behav. Res. Methods.

[20] Paul S., Peter A., Pietrobon N., Hämmerle C.H.F. (2002). Visual and spectrophotometric shade analysis of human teeth. J. Dent. Res..

[21] Zaher A.R., Abdalla E.M., Motie M.A.A., Rehman N.A., Kassem H., E Athanasiou A. (2012). Enamel colour changes after debonding using various bonding systems. J. Orthod..

[22] Vichi A., Louca C., Corciolani G., Ferrari M. (2011). Color related to ceramic and zirconia restorations: A review. Dent. Mater..

[23] Bollenl C.M., Lambrechts P., Quirynen M. (1997). Comparison of surface roughness of oral hard materials to the threshold surface roughness for bacterial plaque retention: A review of the literature. Dent. Mater..

[24] Vichi A., Fonzar R.F., Goracci C., Carrabba M., Ferrari M. (2018). Effect of Finishing and Polishing on Roughness and Gloss of Lithium Disilicate and Lithium Silicate Zirconia Reinforced Glass Ceramic for CAD/CAM Systems. Oper. Dent..

[25] Jones C.S., Billington R.W., Pearson G.J. (2004). The in vivo perception of roughness of restorations. Br. Dent. J..

[26] Herion D.T., Ferracane J.L., Covell D.A. (2010). Porcelain surface alterations and refinishing after use of two orthodontic bonding methods. Angle Orthod..

[27] Özden A.N., Akaltan F., Can G. (1994). Effect of surface treatments of porcelain on the shear bond strength of applied dual-cured cement. J. Prosthet. Dent..

[28] Mohebi S., Shafiee H.-A., Ameli N. (2017). Evaluation of enamel surface roughness after orthodontic bracket debonding with atomic force microscopy. Am. J. Orthod. Dentofac. Orthop..

[29] Sasany R., Kunt G.E., Koca M.F. (2022). Influence different polishing systems on roughness and colour stability of chairside CAD/CAM blocks with laminate veneer thickness. J. Appl. Biomater. Funct. Mater..

[30] Bourke B.M., Rock W. (1999). Factors Affecting the Shear Bond Strength of Orthodontic Brackets to Porcelain. Br. J. Orthod..

[31] Sarac D., Sarac Y.S., Yuzbasioglu E., Bal S. (2006). The effects of porcelain polishing systems on the color and surface texture of feldspathic porcelain. J. Prosthet. Dent..

[32] Steiner R., Beier U.S., Heiss-Kisielewsky I., Engelmeier R., Dumfahrt H., Dhima M. (2015). Adjusting dental ceramics: An in vitro evaluation of the ability of various ceramic polishing kits to mimic glazed dental ceramic surface. J. Prosthet. Dent..

[33] Flury S., Lussi A., Zimmerli B., Flury A.L.S., Peutzfeldt A. (2010). Performance of Different Polishing Techniques for Direct CAD/CAM Ceramic Restorations. Oper. Dent..

[34] Karan S., Toroglu M.S. (2008). Porcelain Refinishing with Two Different Polishing Systems after Orthodontic Debonding. Angle Orthod..

[35] Anusavice K.J., Phillips R.W. (2003). Phillip’s Science of Dental Materials.

[36] Osorio R., Toledano M., García-Godoy F. (1998). Enamel surface morphology after bracket debonding. ASDC J. Dent. Child..

[37] Martinez-Gomis J., Bizar J., Anglada J., Samsó J., Peraire M. (2003). Comparative evaluation of four finishing systems on one ceramic surface. Int. J. Prosthodont..

[38] Kou W., Molin M., Sjogren G. (2006). Surface roughness of five different dental ceramic core materials after grinding and polishing. J. Oral Rehabil..

[39] Oz A.Z., Oz A.A., Ural C., Kaleli N., Duran İ. (2023). Effectiveness of surface polishing after debonding of metal brackets from different CAD-CAM materials. J. Orofac. Orthoped./Fortschritte Kieferorthopädie.

[40] Siddanna G.D., Dds A.J.V., Fierro P.H., Neiva G.F., Dds D.J.F. (2021). Surface Evaluation of Resilient CAD/CAM ceramics after Contouring and Polishing. J. Esthet. Restor. Dent..

[41] Ryba T.M., Dunn W.J., Murchison D.F. (2002). Surface roughness of various packable composites. Oper. Dent..

[42] Shibasaki N., Cavalli V., Oliveira M.C., Barbosa J.P., Gomes Boriollo M.F., Marcondes Martins L.R. (2021). Influence of Surface Treatment on the Physical Properties and Biofilm Formation of Zirconia-Reinforced Lithium Silicate Ceramics: In Vitro Trial. Int. J. Prosthodont..

[43] Matzinger M., Hahnel S., Preis V., Rosentritt M. (2018). Polishing effects and wear performance of chairside CAD/CAM materials. Clin. Oral Investig..

[44] Lee J.Y., Jang G.W., Park I.I., Heo Y.R., Son M.K. (2019). The effects of surface grinding and polishing on the phase transformation and flexural strength of zirconia. J. Adv. Prosthodont..

[45] Sampaio-Fernandes M., Almeida P.J., Portugal J., Martins R.C., Figueiral M.H. (2022). Effectiveness of ceramics chair-side polishing—2D and 3D roughness evaluation. Rev. Port. Estomatol. Med. Dentária Cir. Maxilofac..

[46] Jum’ah A.A., Brunton P.A., Li K.C., Waddell J.N. (2020). Simulated clinical adjustment and intra-oral polishing of two translucent, monolithic zirconia dental ceramics: An in vitro investigation of surface roughness. J. Dent..

[47] Ruyter I., Nilner K., Möller B. (1987). Color stability of dental composite resin materials for crown and bridge veneers. Dent. Mater..

[48] Johnston W., Kao E. (1989). Assessment of Appearance Match by Visual Observation and Clinical Colorimetry. J. Dent. Res..

[49] Wee A.G., Monaghan P., Johnston W.M. (2002). Variation in color between intended matched shade and fabricated shade of dental porcelain. J. Prosthet. Dent..

[50] Sarıkaya I., Hayran Y. (2018). Effects of polishing on color stability and surface roughness of CAD-CAM ceramics. Meandros Med. Dent. J..

[51] Seghi R., Hewlett E., Kim J. (1989). Visual and Instrumental Colorimetric Assessments of Small Color Differences on Translucent Dental Porcelain. J. Dent. Res..

[52] Öngül D., Şermet B., Balkaya M.C. (2012). Visual and instrumental evaluation of color match ability of 2 shade guides on a ceramic system. J. Prosthet. Dent..

[53] Khashayar G., Dozic A., Kleverlaan C., Feilzer A. (2012). Data Comparison between Two Dental Spectrophotometers. Oper. Dent..

[54] Moradi M., Moradinezhad M., Shamohammadi M., Hormozi E., Ghorani A. (2018). Porcelain color alteration after orthodontic bonding using three different surface preparation methods. Dent. Res. J..

[55] Ameli N., Talaei R., Saeedi S., Ghorbani R., Askari B.A. (2019). Effect of two polishing systems on color and surface roughness of feldspathic porcelain following orthodontic bracket debonding and composite resin removal. APOS Trends Orthod..

